# Case Report: *MYO5B* Homozygous Variant c.2090+3A>T Causes Intron Retention Related to Chronic Cholestasis and Diarrhea

**DOI:** 10.3389/fgene.2022.872836

**Published:** 2022-05-30

**Authors:** Yu Zheng, Yuming Peng, Shuju Zhang, Hongmei Zhao, Weijian Chen, Yongjia Yang, Zhengmao Hu, Qiang Yin, Yu Peng

**Affiliations:** ^1^ First Department of General Surgery & Pediatrics Research Institute of Hunan Province, Hunan Children’s Hospital, Changsha, China; ^2^ Department of Gastroenterology and Nutrition, Hunan Children’s Hospital, Changsha, China; ^3^ Department of Pathology, Hunan Children’s Hospital, Changsha, China; ^4^ Center for Medical Genetics & Hunan Key Laboratory of Medical Genetics, School of Life Sciences, Central South University, Changsha, China

**Keywords:** microvillus inclusion, RNA splicing, intron retention, minigene assay, *MYO5B*, diarrhea, cholestasis

## Abstract

**Background:** Biallelically mutated *MYO5B* is associated with microvillus inclusion disease (MVID, MIM: 251850), cholestasis, or both. This study aims at validating the splicing alteration and clinical features of an intron variant for diagnosis.

**Case Presentation:** A homozygous variant of *MYO5B*, NM_001080467.2:c.2090+3A > T (NP_001073936.1:p.?) in intron 17, was identified in a patient suffering from chronic cholestasis and diarrhea. Functional validation showed that this variant caused 185 bp of intron retention in its mRNA and was predicted to present a premature translation termination site for myoVb (p.Arg697fs*47) in the head motor domain. In addition, bowel biopsy revealed decreased microvilli and local lesions of microvillus inclusion in the duodena of the patient. The patient was presented with neonatal cholestasis leading to cirrhosis, intractable diarrhea, cholelithiasis, hepatic cyst, corneal opacity, and failure to thrive.

**Conclusion:** Our study demonstrated an intronic homozygous variant of *MYO5B* that affected an intron, subsequently altering splicing and leading to combined cholestasis and MVID. Our results further supported the underlying genotype–phenotype correlations and extended clinical practices toward its diagnosis and management.

## Introduction

*MYO5B* encodes myosin Vb protein, which functions as an actin-based molecular motor and plays an essential role in intracellular trafficking and plasma membrane recycling (Johnston et al., 1991; Lapierre et al., 2001; Wu et al., 1998). It is widely expressed in multiple tissues, including the liver, intestine, and brain. *MYO5B* dysfunction was first reported in 2008 to be related to autosomal recessive microvillus inclusion disease (MVID, MIM: 251850) (Erickson et al., 2008; Muller et al., 2008). MVID is characterized by intractable diarrhea in infants with enterocyte brush border defects or congenital microvillus atrophy (Dhekne et al., 2018; Ruemmele et al., 2006). It was also described as the onset of intractable, life-threatening watery diarrhea during infancy (MIM phenotype: diarrhea-2 with microvillus atrophy). Knockdown of *MYO5B* reproduced MVID phenotypes in both intestinal cell lines and animal models, supporting the underlying mechanism (Carton-Garcia et al., 2015; Ruemmele et al., 2010; Schneeberger et al., 2015; Sidhaye et al., 2016; Weis et al., 2016). Later in 2017, Gonzales et al. (2017) and Qiu et al. (2017) found that *MYO5B* deficiency was associated with previously indeterminate forms of progressive familial intrahepatic cholestasis (PFIC). However, the reported patients did not develop MVID. According to the records of patients with *MYO5B* variants and their clinical phenotypes from the International MVID Patient Registry website (https://www.mvid-central.org; accessed March 24 2022; van der Velde et al., 2013; van der Valde et al., 2019), ClinVar (accessed March 24 2022; https://www.ncbi.nlm.nih.gov/clinvar) and the latest publications, about 540 variants of *MYO5B* have been reported, of which 130 were associated with MVID, PFIC, or mixed MVID-PFIC (Aldrian et al., 2021; Wang et al., 2021). As the spectra of genotypes and phenotypes expand, the underlying genotype–phenotype correlations need to be fully understood.

**FIGURE 1:**
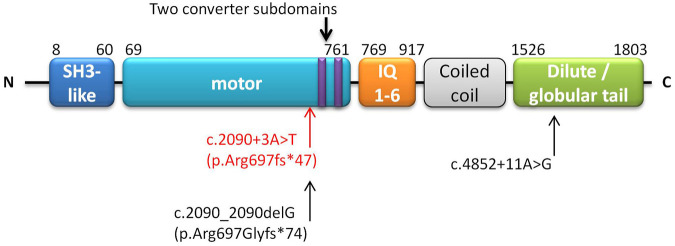
Schematic of domains of the *MYO5B* gene. The annotation is based on UniProt (https://www.uniprot.org/uniprot/Q9ULV0). The homozygous NM_001080467.2:c.2090+3A > T (p.Arg697fs*47) found in this study is marked in red. Another patient harbored c.2090_2090delG (p.Arg697Glyfs*74) (close to our reported variant), along with c.4852 + 11A > G which is marked in black (Qiu et al., 2017).

*MYO5B* defects can cause various phenotypes affecting cells from enterocytes to hepatocytes, and the disease-causing genotypes involve different variants associated with different protein domains. The myoVb protein has five domains or regions: myosin N-terminal SH3-like, myosin motor, isoleucine-glutamine calmodulin-binding (IQ) 1-6, coiled-coil, and dilute (also called globular tail) (https://www.uniprot.org/uniprot/Q9ULV0) (Figure 1). Biallelic mutations in the motor domain can cause aberrant localization of bile canalicular proteins and subapical accumulation of bile salt export pumps in hepatocytes (Overeem et al., 2020; Hess et al., 2021). However, neither a truncated myoVb without the globular tail domain nor a complete deficiency could cause mislocalization of canalicular proteins in hepatocytes (Overeem et al., 2020). On the other hand, in enterocytes, truncated myoVb or total depletion of myoVb causes subapical clusters of aberrant recycling endosomes and microvillus inclusion (Hess et al., 2021). In 2021, Aldrian et al. reviewed and speculated that the loss of function (LoF) variation could lead to MVID, and missense or variations causing aberrant *MYO5B* expression would lead to PFIC or mixed MVID + PFIC (Aldrian et al., 2021). PFIC is associated with aberrations in residual function of the full-length myoVb expressed in hepatocytes and its protein-protein interactions. Conversely, myoVb expressed without residual function is associated with mixed MVID + PFIC (Aldrian et al., 2021).

Here, we report a homozygous intronic variant, NM_001080467.2:c.2090+3A > T, which is located in the motor domain of *MYO5B*. This study aims to verify the functional impact and phenotypic changes of this intronic variant. The variation was transfected to MCF-7 and HEK293T cells to validate the RNA products. We also performed immunohistochemistry and electron microscopy to detect the histological and pathological changes in the mucosa of the patient’s bowel. Through functional and phenotypic analysis, we further confirmed the correlation between the new intron variant and the phenotypes. It would facilitate clinical practice by expanding our understanding of genotype–phenotype correlations for the diagnosis, management, and the prevention of *MYO5B*-associated diseases.

## Case Presentation

A seven-year-old Chinese Han girl was admitted to Hunan Children’s Hospital in China in 2019 having intractable pruritus, chronic diarrhea, corneal opacity, and short stature. By detailed examination, she had decompensated cirrhosis, portal hypertension, cholelithiasis, hepatic cysts, splenomegaly, encephaloedema, proteinuria, malnutrition, and anemia. Her medical history included prolonged jaundice in the first two months of her life. She has experienced loose stools at least three times a day since birth. When she was six months old, she had severe diarrhea and was hospitalized in the pediatric intensive care unit (PICU). Two years later, she was admitted to the PICU again because of bloody stool. Later, she was admitted to the PICU twice, after four months and six months, because of severe diarrhea. Her liver function was abnormal since birth and was reported to have progressive cholestasis with increased direct bilirubin (13.4–29 μmol/L), aspartate aminotransferase (AST, ∼105.1 U/I), and total bile acid (normal at six months of age and gradually increased to 181.2 μmol/L when six years old). A growth delay was observed from the age of six months. Pruritus and yellow skin manifested at the age of two years. At the age of five years, her left eye developed corneal opacity, which subsequently resulted in blindness; she developed corneal opacity in her right eye at the age of six years. At the time of writing this report, she was nine years old and had alleviated diarrhea with unshaped stools approximately three times per day. Supplementary Table S1 summarizes the history of her clinical phenotypes.

Her parents declared that their marriage was not consanguineous, but they were born in the same small district in the central south of China (Yiyang, Hunan Province).

## Methods

### Exome Sequencing and Bioinformatic Analysis

After a hepatopathy-targeted gene panel test returned negative results, trio-based clinical exome sequencing was performed. Genomic DNA was extracted from peripheral blood samples of the patient and her parents. DNA libraries were prepared to capture coding sequences and known pathogenic non-coding regions of over 4,000 human genes recorded in the OMIM (Amberger et al., 2015) and ClinVar (Landrum et al., 2016) databases. The captured libraries were then sequenced using the Illumina HiSeq X Ten system (Illumina, San Diego, California, United States) to generate 2 × 150 bp reads. The average sequencing depth of the three samples was approximately 343 X each. In each sample, 99.7% of the target regions were covered by more than 20X. NextGENe (version: 2.4.1.2, SoftGenetics, LLC, United States) software was employed to polish reads and align them to the human reference genome (version: GRCh37) to call SNPs, InDels, and CNVs, and annotate variants. Subsequently, all detected variants were interpreted and classified according to the American College of Medical Genetics and Genomics (ACMG) guidelines (Richards et al., 2015). We used human phenotype ontology (HPO) keywords to describe patient phenotypes. We then prioritized pathogenic, likely pathogenic, and variants with uncertain significance in associated genes relevant to the patient’s phenotypes. Then, Sanger sequencing was performed to validate the variant with the highest priority in the family.

### Minigene Splicing Assay

We performed a minigene splicing assay to validate whether this variant had splice alterations in mRNA transcripts *in vitro*. The minigene pEGFP-C1-MYO5B-wt/mut was constructed by inserting the whole genomic sequence from exon 17 to exon 18 (including intron 17) into pEGFP-C1 (Supplementary Figure S1) and pcDNA3.1, respectively. We designed two pairs of primers: 282104-MYO5B-F and 283709-MYO5B-R, and 282511-MYO5B-F and 283355-MYO5B-R (Supplementary Table S2). Nested PCR was performed at 57°C using 0.5 µg of the patient’s gDNA. Using the second-round product of the nested PCR as a template, wild-type or mutational (wt/mut) segments were generated by employing the following steps: 1) primers GFP-MYO5B-HindIII-F and MYO5B-intron17-R were used to obtain the 629 bp-long segment 1 of pEGFP-C1; 2) primers MYO5B-intron17-F and GFP-MYO5B-BamHI-R were used to obtain the 290 bp-long segment 2 of pEGFP-C1; 3) segments 1 and 2 were recycled and mixed in a 1:1 ratio to perform PCR. We then obtained a final segment of pEGFP-C1 with a length of 883 bp. Subsequently, enzyme digestion, recovery, ligation, transformation, and colony PCR identification were performed for the vector pEGFP-C1 and DNA fragments (the reaction conditions and the results of gel electrophoresis are shown in Supplementary Figure S2). The wt/mut sequences were validated using Sanger sequencing. The vector pcDNA3.1 was constructed similar to pEGFP-C1.

**FIGURE 2:**
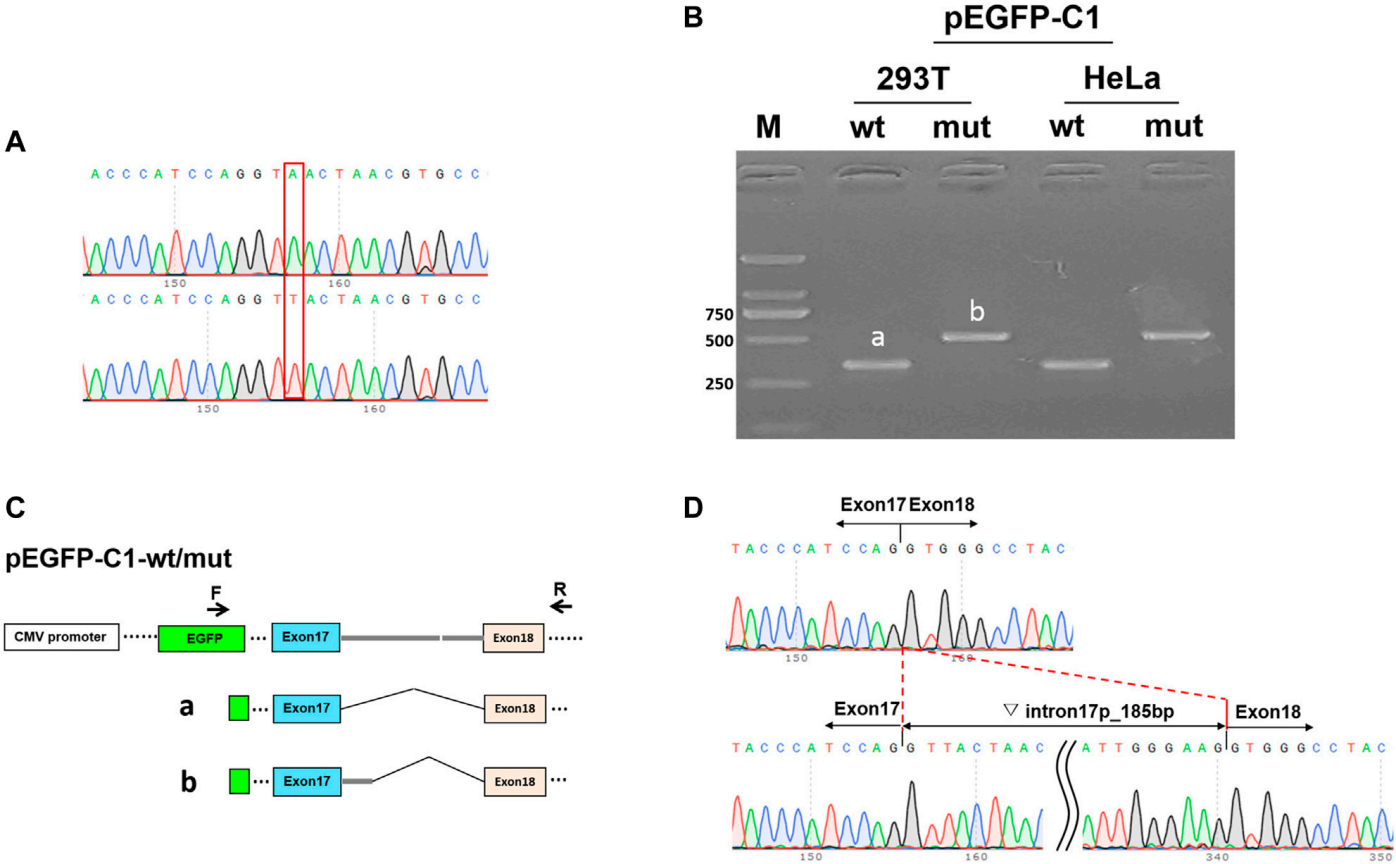
Minigene splicing assay in HEK293T and HeLa cells. **(A)** Sequences of the vector pEGFP-C1. Up: wild-type (wt), down: mutant (mut). **(B)** Image representing the results of gel electrophoresis shows the amplified transcript during RT-PCR corresponding to cDNAs isolated from HEK293T and HeLa cells. a: wt, b: mut. **(C)** pEGFP-C1-wt/mut construction and schematic diagram of the splice. **(D)** Sequence at the site of mutation in pEGFP-C1-MYO5B-wt (up) and pEGFP-C1-MYO5B-mut (down).

Subsequently, the constructed vectors pEGFP-C1-MYO5B-wt/mut (sequences shown in Figure 2A) were transiently transfected into MCF-7 and HEK293T cells using low-toxicity Lipofectamine following the manufacturer’s instructions (Life Technologies, Carlsbad, CA, United States). The product was recycled after 36 h. Total RNA was isolated using TaKaRa TRIzol (RNAiso PLUS) and then reverse transcribed to generate cDNA. Reverse transcription-polymerase chain reaction (RT-PCR) was performed using this cDNA as a template, and bidirectional sequencing using an ABI 3130 DNA analyzer was done (Applied Biosystems, Foster City, CA, United States). Simultaneously, we tested pcDNA3.1-MYO5B-wt/mut following similar steps. Figure 2C shows the scheme of splice region sequencing in the minigene splicing assay.

### Protein Sequence and Structure Prediction

zWe predicted the protein sequence using the generated mutant cDNA sequence based on the minigene splicing assay. Lasergene EditSeq Pro software (version 7.1.0; DNASTAR, Inc., United States) was used to translate the mutant cDNA sequence into an amino-acid sequence. The 3D crystal structure of the motor domain of wild-type myosin-Vb (PDB ID: 1OE9) was displayed using Cn3D (version: 4.3.1). For homology modeling, the mutant sequence was loaded into the SWISS-MODEL (“SWISS-MODEL. https://swissmodel.expasy.org/,” accessed June 25 2021). Structural modification of the mutant myoVb protein was predicted using Swiss-Pdb Viewer software (SWISS-MODEL. https://swissmodel.expasy.org/).

### Immunohistochemistry and Electron Microscopy

Biopsies of the patient’s duodenum, small intestine, large intestine, and colon were performed. Tissue specimens were fixed in 4% acetic formalin, embedded in paraffin, stained with periodic acid-Schiff (PAS), and immunostained with antibodies against CD10 (ZSGB-BIO, Zhongshan Jinqiao Biotechnology Co. Ltd., Beijing, China). Duodenum specimens were fixed in glutaraldehyde, cut into 0.2 cm^3^ sections, and then scanned using an electron microscope.

## Results

### Biallelic c.2090+3A > T in Intron 17

Exome sequencing identified a novel biallelic variation NM_001080467.2:c.2090+3A > T (NP_001073936.1:p.?) in the *MYO5B* gene of the patient; both her parents were found to carry a monoallelic variant of c.2090+3A > T each (Supplementary Table S3). This variant was absent from the gnomAD (Wang et al., 2020) and ClinVar (accessed June 25 2021) databases. It was revealed to be located in the motor domain of the myoVb protein (Figure 1), which is a mutational hotspot region. This variant was predicted to be splice altering and deleterious by bioinformatics software dbscSNV (Ada score = 1, RF score = 0.9) (Liu et al., 2016), SpliceAI (score = 0.85) (Jaganathan et al., 2019) and Trap (version 3.0, score = 0.895) (accessed May 23, 2021; Gelfman et al., 2017). In addition, the patient’s phenotype was highly specific to MVID-PFIC, which indicated a single genetic etiology. According to the ACMG criteria (Richards et al., 2015) and varsome query (https://varsome.com/; accessed May 10, 2021; Kopanas et al., 2019), this variant was classified as likely pathogenic (PM1, PM2, PP3, and PP4).

### Intron Retention of 185 bp

In the minigene splicing assay for c.2090+3A > T, RT-PCR detected a longer product of pEGFP-C1-MYO5B-mut (slightly over 500 bp) than of the wild-type pEGFP-C1-MYO5B-wt (335 bp) in the transfected MCF-7 cells, and similarly, a longer product of pcDNA3.1-MYO5B-mut (over 500 bp) than of the wild-type pcDNA3.1-MYO5B-wt (403 bp) in the transfected HEK293T cells (Figure 2B). Sanger sequencing demonstrated that the splicing model of the wild type (pEGFP-C1-MYO5B-wt and pcDNA3.1-MYO5B-wt) was Exon17 (87 bp)-Exon18 (112 bp), while that of the mutant type (pEGFP-C1-MYO5B-mut and pcDNA3.1-MYO5B-mut) was Exon17 (87 bp)-▽intron17 (185 bp)-Exon18 (112 bp) (Figure 2D). Thus, the expressed mutant transcripts harbored an otherwise spliced intron of 185 bp from the 5′ strand of intron 17 in the transfected cells (Figure 2).

### Truncated myoVb at the Motor Domain in Prediction

Based on the mutant sequence of the transcripts, the retention of the intron sequence was predicted to be translated into 44 amino acids and then terminated. It revealed that the variant *MYO5B*:c.2090+3A > T would generate p. Arg697fs*47 in the protein. The translation terminated near the C terminal in the motor domain (Figure 1). In the wild-type myoVb, the motor domain ended at 761 aa (Figure 1). Supplementary Figure S3 shows the structure of the motor domain of wild-type myosin V and the predicted structure of the mutant myoVb. This variant occurs at the upstream of two converter subdomains (703–713 aa and 734–749 aa, Figure 1 and Supplementary Figure S3), and play a role in amplifying the conformational changes of the motor domain (Houdusse et al., 1999).

### Mixed MVID-PFIC With Decreased Microvillus

**FIGURE 3:**
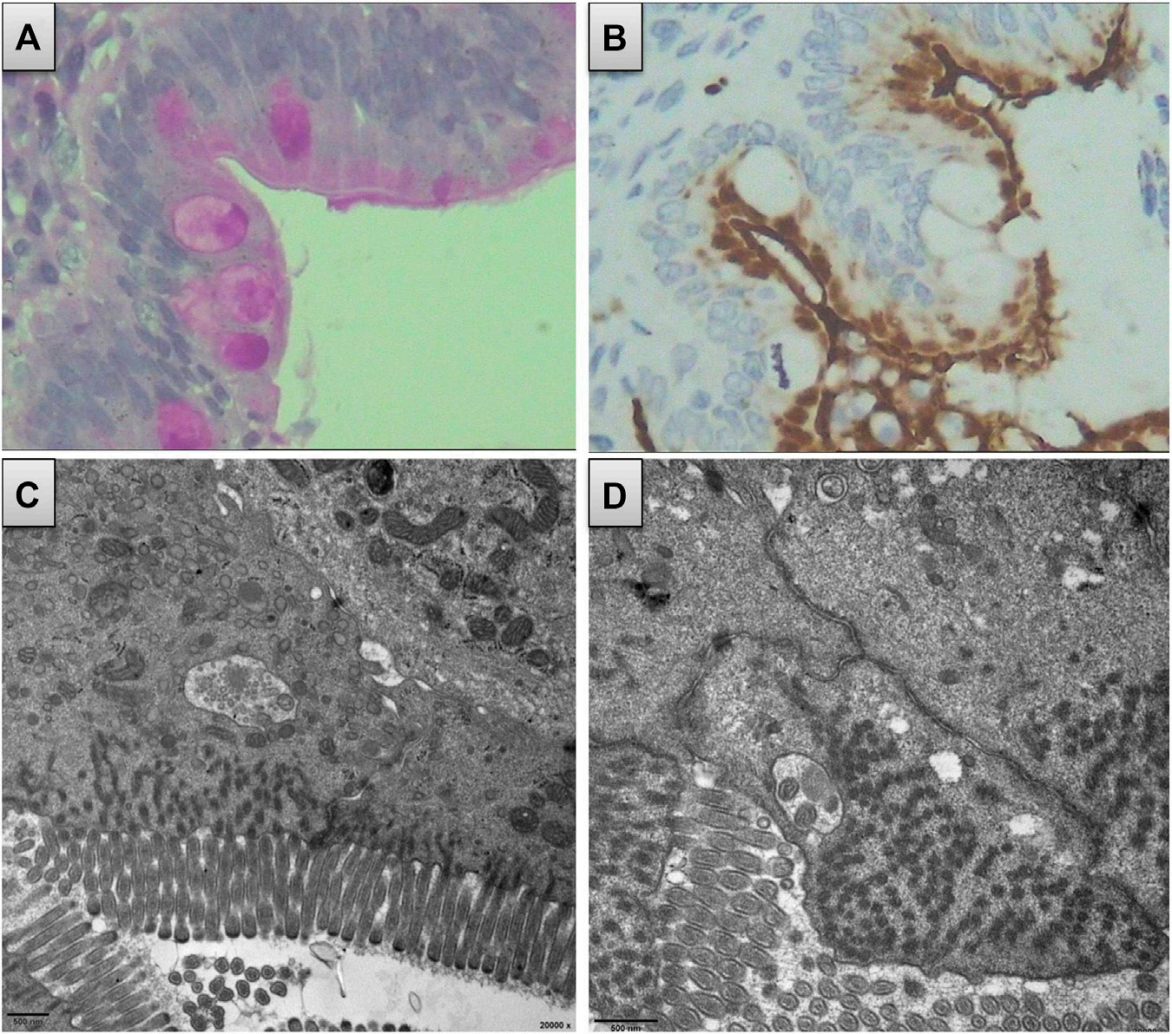
Duodenum biopsy revealed decreased microvillus and local lesion of microvillus inclusion. **(A)** Decreased microvillus and obscure brush border appear in immunohistochemistry using PAS. **(B)** Obscure brush border of microvillus and internalized appearance using CD10 staining. **(C)** and **(D)** Local lesion of microvillus inclusion from the electron microscope.

Both immunohistochemistry and electron microscopy revealed decreased microvilli and local lesions of microvillus inclusions in the mucosa of the patient’s duodenum (Figure 3). No abnormal microvilli were observed in the small bowel, large bowel, or colon specimens. As no liver biopsy was available, we could not observe any pathological changes in the liver. Nevertheless, the patient had a history of disrupted liver function with prolonged jaundice and progressive cholestasis, along with liver cirrhosis, which progressed to end-stage disease. In addition, she had chronic malnutrition and delayed development. Therefore, the presence of a mixed MVID-PFIC was confirmed. Altogether, the c.2090+3A > T variant in *MYO5B* was classified as a likely pathogenic one (criteria matched PS3, PM1, PM2, PP3, and PP4).

### Phenotype Development and Clinical Management

The female patient under investigation in this study has had progressive cholestasis since birth. Upto the age of 7 years, she manifested decompensated cirrhosis, portal hypertension, cholelithiasis, hepatic cysts, splenomegaly, encephaledema, corneal opacity, malnutrition, anemia, growth delay, and short stature. Since the patient was 5 years old, cholestyramine and ursodeoxycholic acid were administered daily to alleviate liver cirrhosis and pruritus. In addition, bifid tetragenous viable bacterial tablets, vitamin A, and vitamin D were routinely administered to maintain the intestines and provide nutritional supplements. Cefdinir dispersible tablets have also been used to control bacterial infections and to prevent sepsis. The patient refused liver transplantation. At the time of report preparation, the patient was alive and had mild diarrhea.

## Discussion

This study identified a homozygous intronic variation, c.2090+3A > T, in *MYO5B* in a female patient with mixed MVID-PFIC. The minigene splicing assay revealed that this variant caused a 185 bp retention of intron 17 in the transcripts, which was predicted to generate the following change in the amino acid sequence: p. Arg697fs*47. Consequently, it led to a premature termination of translation near the end of the motor domain of myoVb. Moreover, bowel biopsies verified a decrease in microvilli and local lesions of microvillus inclusion in the duodenum. The patient was presented with severe cholestasis, and chronic diarrhea was alleviated from the severe conditions occasionally experienced in her early life. Combining the patient’s clinical features and literature review, the genotype–phenotype relationship of this newly identified splicing variant was established.

This variant causes the loss of the two downstream converter subdomains, six IQ light chains, coiled-coil, and the globular tail (dilute) domain. Recently, an analysis of 130 *MYO5B*-associated patients found that non-null variants were related to the presence of PFIC, whereas null variants increased the severity of intestinal manifestations and cholestasis (Wang et al., 2021). Here, the myoVb mutant with the remaining motor domain would act as the binding actin to utilize ATP hydrolysis to generate directed movement toward the plus end along the actin filaments (Trybus 2008). In a previous study, another myoVb mutant, p. Arg363*, with a truncated incomplete motor domain, was found to be expressed in hepatocytes with normal polarity and canalicular protein localization (Overeem et al., 2020). However, the apical bile transporter ABCC2/MRP2 was mislocated intracellularly, leading to cholestasis in hepatocytes (Overeem et al., 2020; Aldrian et al., 2021). In addition, truncated myoVb protein molecules were aberrantly localized in enterocytes with Rab11 and syntaxin abnormally bound to them (Hess et al., 2021). The microvilli were generally shorter but focally had deeper rootlets and no brush border than the wild type, and microvillus inclusions were found sporadically in the epithelium of the duodenal biopsies (Hess et al., 2021). Aldrian et al. (2021) proposed that these kinds of truncated proteins would cause decreased apical brush border, subapical secretory granules, and mislocated apical proteins. In this study, the patient presented with persistent cholestasis and moderate-to-mild diarrhea, the phenotype of which was consistent with those reported by previous studies and confirmed the genotype–phenotype hypothesis.

A variation NM_001080467.2:c.2090_2090delG, close to our reported variation of c.2090+3A > T, has been reported previously, where it was predicted to generate a p. Arg697Glyfs*74 variation in its protein (Qiu et al., 2017). The patient harbored this monoallelic variant along with another variant, NM_001080467.2:c.4852 + 11A > G, forming a compound heterozygous variation presenting cholestasis (or PFIC) alone. c.2090_2090delG is in the motor domain, the same as our reported homozygous variant, and c.4852 + 11A > G is in the globular tail domain near the N-terminal myoVb (Figure 1). The compound variants were proposed to produce a myoVb mutant with residual function, causing PFIC but without any intestinal disorders.

In this study, the patient had previously undergone a gene-based and then a panel-based genetic testing but had received negative results. Exome sequencing was the third-tier genetic testing that finally revealed *MYO5B* to be the disease-causing gene. Interestingly, cholestasis is a recently diagnosed phenotype that was not recognized previously related with *MYO5B* (Qiu et al., 2017); till then, infantile diarrhea was the only recognized phenotype. The patient did not obtain a precise diagnosis until she was 6 years old. The congenital disorder led to severe malnutrition, severe growth delay, and almost complete blindness eyes in her late life. One of the reasons for this delayed diagnosis is the developing molecular testing technology whose clinic application is slow, especially in developing countries or less developed areas. Another reason is that *MYO5B*-associated diseases and their phenotypes are not fully understood. The disease-targeted gene panel might not include the associated genes over time. Whole-exome sequencing and whole-genome sequencing will generate more incidental findings, and routine reanalysis will help find more genotype–phenotype correlations and decrease the turn-around time in the clinic. For this patient, early diagnosis and intervention could have improved the quality of her forthcoming life.

This study has certain limitations. First, no liver biopsies were available, and thus we could not further examine the pathological changes in the liver in association to the detected variant. Other limitations include the lack of cDNA analysis on the mRNA extracted from the patient bowel tissue as well as the lack of alteration detection of weight and structure in the mutant protein product. Further studies regarding the interaction between mutant proteins and other proteins are required.

## Conclusion

In this study, we identified a homozygous variant, c.2090+3A > T, in the *MYO5B* gene and validated its splicing alteration, resulting in intron retention and the subsequent premature translation termination. With an incomplete motor domain of the truncated myoVb, the patient had a mixed phenotype with intrahepatic cholestasis and MVID. Pathology further confirmed decreased microvilli and local lesions of microvillus inclusion in the duodenum. Thus, our study provides new evidence supporting the previous hypothesis of the genotypic–phenotypic spectrum of *MYO5B* and the clinical implications associated with disease diagnosis and management.

## Data Availability

The datasets presented in this study can be found in the supplementary materials and online repositories. The reported variant was submitted to ClinVar (https://www.ncbi.nlm.nih.gov/clinvar/variation/1184992/).

## Abbreviations

MVID, microvillus inclusion disease; PFIC, progressive familial intrahepatic cholestasis; BSEP, bile salt export pump; LoF, loss of function.
