# Supplementary material for: Case Report: MYO5B Homozygous Variant c.2090+3A>T Causes Intron Retention Related to Chronic Cholestasis and Diarrhea
Source: Front Genet. 2022 May 30;13:872836. doi: 10.3389/fgene.2022.872836 (PMC9189387; doi:10.3389/fgene.2022.872836)
Supplement: Supplementary file 3 [file DataSheet1.pdf]

Supplementary Figures

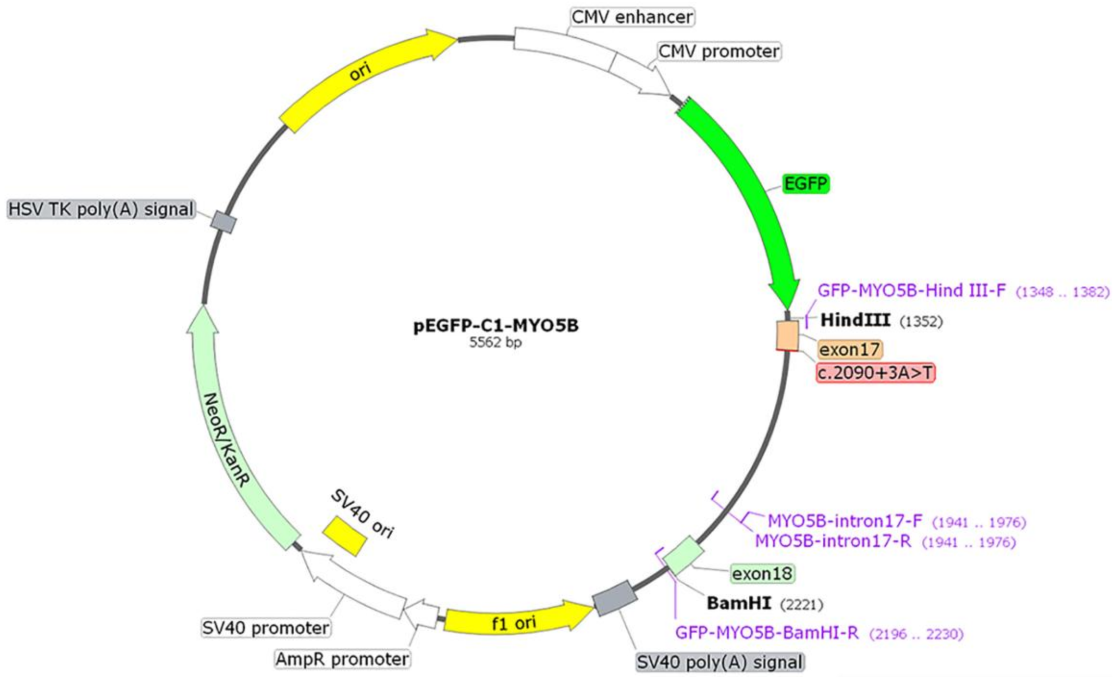

Figure S1 | The strategy of constructing pEGFP-C1-MYO5B-wt/mut using vector pEGFP-C1 in minigene assay. The whole genomic sequence from Exon 17, intron 17 to Exon 18 was inserted into vector pEGFP-C1. The variant c.2090+3A>T shows in red. The location of the designed primers shows in purple.

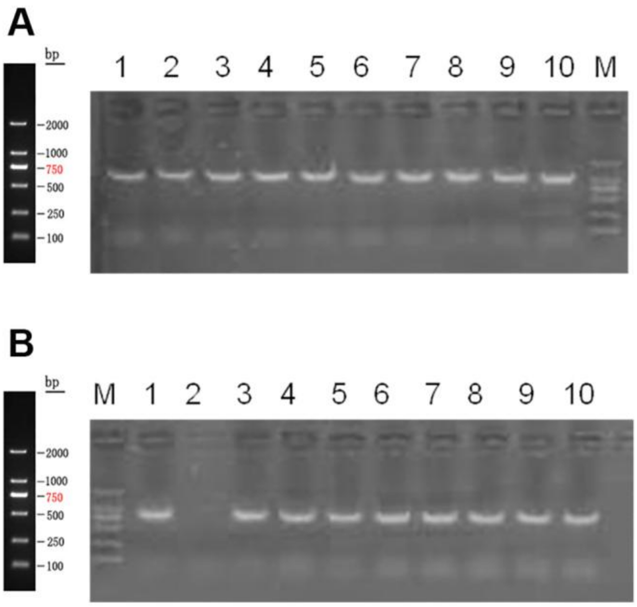

Figure S2 | Gel electrophoresis results of bacterial colony PCR and used reaction conditions. **(A)** Results of bacterial colony PCR for pEGFP-C1-wt/mut using primers pEGFP-C1-5' and

MYO5B-test-R. Lane 1–5: pEGFP-C1–MYO5B–wt bacterial colony, Lane 6–10: pEGFP-C1–MYO5B–mut bacterial colony. Lane M: 2000bp ladder marker. **(B)** Results of bacterial colony PCR for pcDNA3.1-wt/mut using primers MYO5B-test-F and pcDNA3.1-R. Lane 1–5: pcDNA3.1-MYO5B-wt bacterial colony, Lane 6–10: pcDNA3.1-MYO5B- mut bacterial colony, Lane M: 2000bp ladder marker. Reaction conditions for enzymatice digestion: 3 µl 10×NEB buffer, 0.6 µl Enzyme 1, 0.6 µl Enzyme 2, 500 ng / 25 µl Vector/DNA fragment, and 30 µl ddH2O, digested two hours in 37℃. Reaction conditions for ligation: 1 µl 10×ligase buffer, 7 µl digested DNA fragment (wt/mut), 1 µl digested vector, and 1 µl ligase reacted overnight in 4℃ and then transformed to DH5a competent condition.

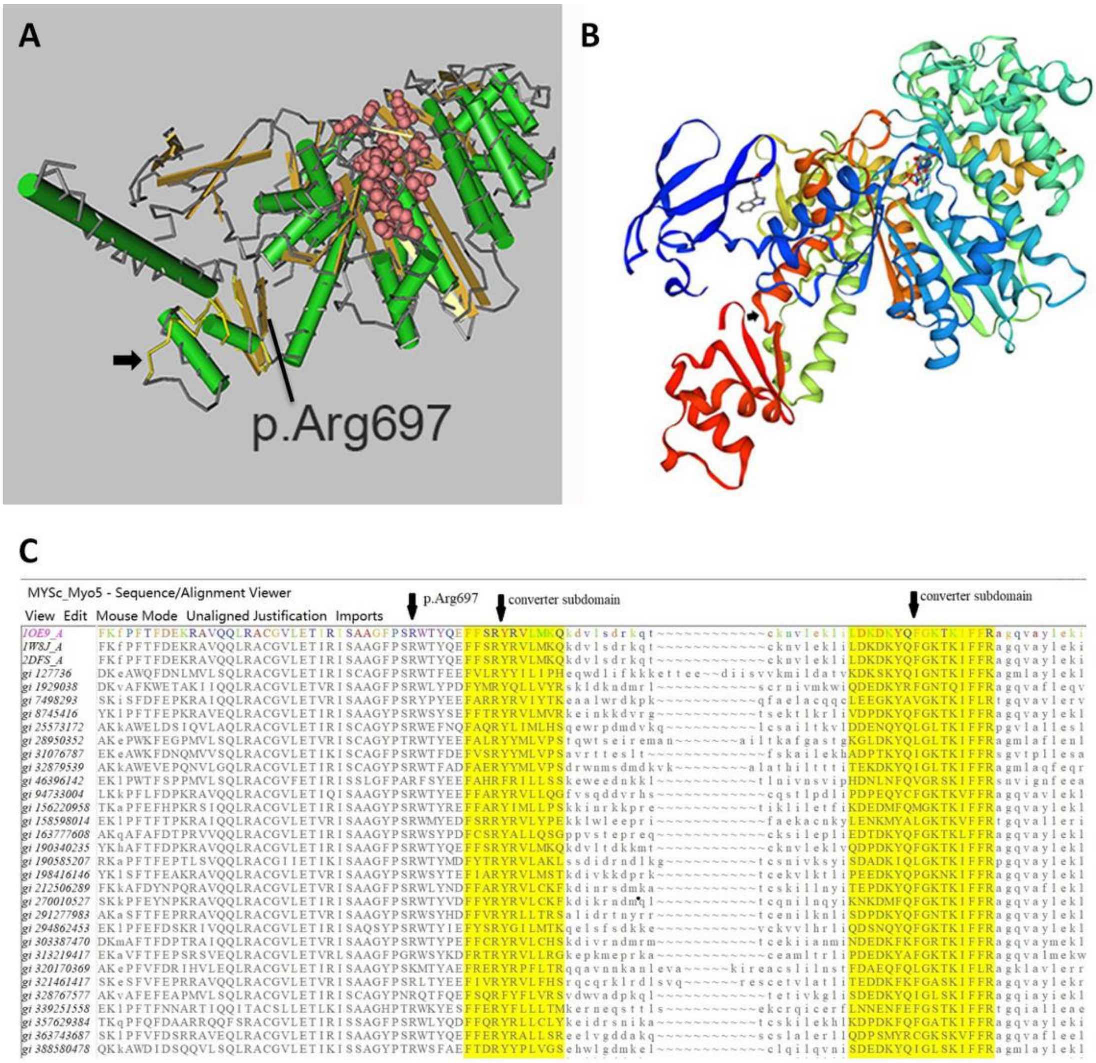

Figure S3 | Predicted structure of the motor domain of myoVb and its sequence alignment. (**A**) Structure of the motor domain of wild myosin V. p.Arg697 (marked using black arrow) is the closest amino acid to the c.2090+3A>T variant. (**B**) The predicted structure of the motor domain of mutant myoVb. The black arrow indicates the position of p.Arg697 next to c.2090+3A>T. (**C**) Sequence alignment viewer shows the positions of p.Arg697 and the downstream two converter subdomains (highlighted in yellow) in the motor domain of myosin V (PDB: 1OE9).
